# The Emotional State of Second-Language Learners in a Research Writing Course: Do Academic Orientation and Major Matter?

**DOI:** 10.3390/bs13110919

**Published:** 2023-11-10

**Authors:** Maura A. E. Pilotti, Arifi Waked, Khadija El Alaoui, Samia Kort, Omar J. Elmoussa

**Affiliations:** 1 Department of Sciences and Human Studies, Prince Mohammad Bin Fahd University, Al Khobar 31952, Saudi Arabia; amohammedwaked@pmu.edu.sa (A.W.); lalaoui@pmu.edu.sa (K.E.A.); skort@pmu.edu.sa (S.K.); 2 Department of Student Affairs, Prince Mohammad Bin Fahd University, Al Khobar 31952, Saudi Arabia; omar@pmu.edu.sa

**Keywords:** happiness, writing anxiety, learning orientation, grade orientation, Middle East

## Abstract

This study examined whether differences exist in the emotional state of students whose approach to undergraduate courses is either preferentially learning-oriented or grade-oriented. It focused on an understudied population of female college students of Saudi Arabian descent who were enrolled in a challenging writing course. Their emotional state was assessed both globally, through the appraisal of their degree of happiness, and locally, through the appraisal of their writing anxiety (a task-specific emotional state). The study contributed to the extant literature by examining whether the association between goal orientation and emotional state, which is predicted by goal orientation theory, could be found in the selected understudied student population. Results illustrate differences between STEM and non-STEM learners. For STEM students, a grade orientation was associated with declining self-reported happiness and increasing writing anxiety. In contrast, for both STEM and non-STEM students, a learning orientation was associated with increasing happiness and declining writing anxiety. Differences existed in the particular type of writing anxiety that was experienced by STEM and non-STEM students. These findings suggest that interventions for students who are struggling academically may need to address personal dispositions if such interventions are to foster subjective well-being (including positive emotions).

## 1. Introduction

According to the theory of goal orientation [1], why (i.e., motives) and how (i.e., actions) a person desires to achieve various objectives define that person’s orientation. Within this theory, orientations are generally conceptualized as enduring dispositions towards engagement. They are conceived as cognitive representations of what the person is trying to do or what the person wants to achieve in a given domain, situation, or task. In academic matters, the particular motives behind students’ academic conduct are believed to be relevant to not only attainment but also well-being [2]. Intrinsically motivated students are those who perform academic activities for the pleasure and satisfaction that can be derived from such activities, such as knowing something new [3]. That is, they prioritize learning over tangible rewards, such as grades. According to Eison et al. [4], a learning orientation is the attitude “held by those students who approach the college experience as an opportunity to acquire knowledge and to obtain educational and personal enlightenment” (p. 2). Extrinsically motivated students are those who engage in academic activities because of the particular tangible outcomes that can arise from such activities [5]. According to Eison et al. [4], grade orientation specifically refers to “students who view obtaining a good course grade, in and of itself, a valid reason for their being and doing in college” (p. 2). In such students, attention is disproportionately devoted to grades instead of learning, including its progression and outcomes [3]. Thus, the appearance of achievement, as manifested by grades, becomes more important than actual learning [6].

Although the primary function of educational institutions is ensuring that students acquire valuable skills and knowledge, grades are overblown metrics of merit. Grades not only are used to measure learning but also serve as necessary commodities for gaining highly desirable rewards (e.g., scholarships). By doing so, educational institutions across the world reinforce a grade orientation in their students. Not surprisingly, the two attitudes, although incompatible, may coexist in learners [7]. First, extrinsic rewards, such as grades, provide information as well as affect so that even for learning-oriented students grades have value. Grades may even serve as a simplistic form of feedback for areas of improvement. Second, different motives may be related to different academic activities. For instance, students may prioritize grades in a compulsory course whose content is judged to be uninteresting or irrelevant. Conversely, the same students may prioritize learning in an elective course whose content is viewed as personally relevant. However, if grade orientation is the overriding disposition, students will pay close attention to their grades even in a course whose content is judged to be personally relevant. They may withdraw from it if they expect their grade point average (GPA) to decline as a result of poor performance. Conversely, if learning orientation is the overriding disposition, students may search for useful and personally relevant content and activities even in what they perceive to be a dull mandatory course.

## 2. Literature Review

The classification of students as either predominantly learning- or grade-oriented often implies differences in academic performance. Grade orientation, for instance, has been associated with students’ lower GPA, scholastic aptitude test (SAT) scores, course test scores, and overall poor academic performance [8,9,10,11]. Poor academic outcomes have been linked to students who view classes as a necessary and unavoidable step in the process of attaining tangible rewards (e.g., a professional certification or suitable employment) [12]. For such students, education is conceived as a means to an end [13]. More pointedly, grade-oriented students’ less desirable academic performance has been attributed to ineffective study habits and high test anxiety [12,14]. Grade-oriented students have also been found to exhibit lower levels of engagement with classmates, which may be detrimental to their ability to perform well on assignments and tests [8,14,15]. Another alleged outcome of grade orientation is that students with such an orientation tend to have a low internal locus of control. That is, they express little confidence in their ability to control success or failure in the classroom [12]. Attributing course performance to luck, chance, or the actions of others (e.g., the instructor) may make grade-oriented students less likely to express proactive actions, such as seeking help from an instructor or teaching assistant or attempting to change study habits deemed ineffective.

Important to note is that evidence supporting differences in academic performance between learning- and grade-oriented students is not homogeneous. For instance, Debicki et al. [16] reported that a learning orientation did not impact academic performance in students from North America. Instead, Lin et al. [17] found learning orientation to be linked to higher grades in students from both North America and East Asia. In Middle Eastern students of Saudi Arabian descent, Pilotti et al. [7] did not find the distinction between grade and learning orientation to be related to academic performance. However, among Kurdish students, Pilotti et al. [18] uncovered gender differences. For females, academic success was related to a learning orientation, whereas for males, no relationship was found between orientation and performance.

In addition to the examination of academic performance in learning- and grade-oriented students, the extant literature offers a handful of studies on the emotional state of such students. However, the evidence regarding the relationship between students’ academic orientation and their emotional state is meager, sometimes anecdotal, and limited to specific disciplines. For instance, in a longitudinal study, Sheldon and Krieger [19] reported an association between first-year law students’ increasing levels of psychological distress and external motives. Larcombe et al. [20] argued that the experience of being a law student may increase one’s grade orientation. Then, they proposed that the latter, an expression of extrinsic motivation, is likely to be related to enhanced psychological distress. Tani and Vines [21] found that law students were more likely than students in other degree programs to be focused on grades and academic attainment. Although Tani and Vines did not directly assess law students’ levels of psychological distress, they speculated that extrinsic motives may be linked to these students’ mental health issues. Among undergraduate students majoring in biological sciences who were taking an introductory physics course, Hall and Webb [22] found that grade orientation was associated with increased anxiety and weakened interest and enjoyment in learning physics. In students enrolled in a management course, Debicki et al. [16] found a positive association between learning orientation and core self-evaluations defined as students’ assessments of their worthiness, competence, and ability. Among students enrolled in a communication course of the general education curriculum, Vallade et al. [23] reported that grade orientation was inversely related to affective learning. The latter was defined as positive feelings that students experience for a particular course, including its content and instructor. Similarly, in an introductory psychology course, Eison [14] found that grade-oriented students were less likely to report positive affect toward either the course or the instructor. They also experienced considerable test anxiety compared to learning-oriented students.

Of course, students’ emotional state may be conceptualized broadly as encompassing the idiosyncrasies of situations and tasks, such as happiness (i.e., a synonym of subjective well-being) [24,25,26]. Alternatively, it may be described as specific to a particular task and situation, such as writing anxiety [27]. The extent to which happiness, as an index of well-being, is linked to different academic motivations (i.e., what makes human beings tick) is not clear, especially in Middle Eastern students. Findings of research on happiness have often portrayed it to lead to positive outcomes, rather than being the result of positive outcomes [28], such as high levels of academic engagement [29] and greater productivity [30]. Yet, Giannetti et al. [31], Bukhari and Khanam [32], and Önder [24] found a rather weak link between academic performance and happiness. Lumontod [33] also reported a weak link between the two variables specifically in freshman students. Instead, Pekrun et al. [34] found robust positive relationships between happiness and variables embodying successful learning, such as self-regulation, motivation (i.e., degree of study interest and effort), learning strategies (e.g., reliance on elaboration), and cognitive resources (i.e., ability to avoid task-irrelevant thinking). Students’ enjoyment measured early in the semester was also found to predict grades at the end of the semester. In contrast, Tuntiwarodom and Potipiti [35] reported a negative relationship between academic performance, as measured by grades, and happiness.

It is unclear whether there is a relationship between academic orientation and anxiety related to a particular skill, such as writing, that is critical to performance in most college courses. Investigations of the relationship between anxiety and writing output have focused on either errors (i.e., performance) or the sources of writing anxiety. Overall, anxious writers compared to less anxious writers have been reported to produce shorter text of inferior quality, including more errors and underdeveloped content and syntax [36,37,38]. Learners attribute writing anxiety to a variety of factors, including linguistic difficulties, inadequate practice, low self-confidence, and fear of writing tests [39]. Students’ overall practice with writing tends to be linked to lower writing anxiety [39,40]. More broadly, Elliot and McGregor [41] reported that the trait of test anxiety is more strongly related to grade orientation than learning orientation. Similarly, Pekrun et al. [34] argued that negative emotions might predict students’ course withdrawals and be significantly more intense in students who drop out compared to students who finish their studies.

## 3. The Aims of the Present Study

Although a variety of student samples have provided evidence that the affective state of grade-oriented and learning-oriented students may differ, to our knowledge, none of these samples includes Middle Eastern students. The present study aims to empirically test whether Middle Eastern students who enter college courses with different academic dispositions or orientations also exhibit different emotional states. The study is also driven by the need to assess the validity of beliefs about students that instructors may have developed through casual observations. Undoubtedly, when scientific evidence is scarce, anecdotes proliferate and solidify into beliefs. The aim here is to ensure that such beliefs are not the byproduct of illusory correlations [42,43]. For instance, in the post-pandemic era, an informal poll of teaching faculty at the Middle Eastern university where the present study is conducted (Saudi Arabia) suggests that educators possess numerous anecdotes of two types of students (see Appendix A). Students who prioritize grades over learning are depicted as being plagued with negative emotions. In contrast, students whose attention is focused on skill and knowledge acquisition are characterized as experiencing negative emotions less frequently. Anecdotes can generate false beliefs about students, which then become resistant to change even in the face of contradictory evidence [44].

The present study rests on the question of whether evidence of differences in the emotional state of learning- and grade-oriented students, collected in the pre-pandemic era, applies to the post-pandemic environment. The pandemic may be considered a destructive (i.e., traumatic) event that altered ingrained habits [45], affective and cognitive states [46], as well as the motives that drive people’s actions [47]. For instance, Copeland et al. [46] found disruptions in students’ behavioral and emotional functioning, including declines in self-reported mood, wellness behaviors, and attention. Jereb et al. [48] reported declines in students’ motivation, learning goals, and attention. Concerning the particular motives that are of interest here, the evidence is mixed if the periods before and during the pandemic are compared. As an example, Daniels et al. [49] found declines in both learning orientation and grade orientation, as well as engagement and perceptions of success. Instead, Smith et al. [50] found an increase in learning orientation, but this was mostly limited to a group of students defined as extraverted.

The aftermath of a destructive event, such as the pandemic, may still linger. If students have preserved their goal preferences during the pandemic, the pre-pandemic distinction between students whose prevailing concern is learning and those whose prevailing concern entails grades will remain valuable. However, the distinction between learning-oriented and grade-oriented students may have become moot if students see learning as the ultimate goal and grades (extrinsic rewards) as useful informational feedback for identifying areas of improvement.

The present study takes place in the post-pandemic era. It specifically examines the extent to which grade or learning orientation is associated with a positive emotion that is synonymous with subjective well-being (i.e., happiness) [24,25,26,51] and a task-specific negative emotion (i.e., writing anxiety) [27]. Through the measurement of these two variables, we wish to capture the extent to which a person may report happiness in general while at the same time exhibiting anxiety when presented with a specific task or situation. In this study, happiness is defined as encapsulating the different evaluations “that people make regarding their lives, the events happening to them, their bodies and minds, and the circumstances in which they live” [52] (p. 400). In contrast to the broad nature of happiness, the variable of writing anxiety is intended to measure students’ self-reported emotional response to the mastering of a skill that is diagnostic of their performance in a challenging course. Thus, it serves as an additional piece of evidence regarding the relationship between students’ predominant academic orientation and their emotional state. For the study, participants are selected from a mandatory research writing course in the general education curriculum that has been reported as considerably challenging in students’ course evaluations. The challenge is perceived to be writing a research report in a second language (English).

It is important to consider that any relationship between academic motivation and affective states in Middle Eastern students is defined by the customs and traditions of the Middle East, which are challenged by the enhanced interconnectedness and interdependence of nations and peoples in the modern globalized world. Globalization brings individualism and accompanying consumerism, largely of Western import, face to face with collectivism, which is a local cultural phenomenon. Individualism and collectivism define a person’s disposition toward oneself and others differently. Individualism refers to attitudes, beliefs, and behaviors that prioritize oneself and close family members. Instead, collectivism refers to a sense of belonging and loyalty to larger in-groups or collectives [53,54]. In the Middle East of today, the value of individual independence coexists with that of collective interdependence [55]. Conceptions of good life and well-being, including happiness, are in essence negotiations between the pleasure of individual autonomy and personal growth, and the honor and satisfaction of contributing to the community to which one belongs [56].

The coexistence of collectivistic and individualistic motives is driven by the programmatic plans of some Middle Eastern countries. For instance, in Saudi Arabia, the 2030 Vision is a socio-economic plan to transform a fossil-fuel economy into one that is knowledge- and service-based. The entire plan rests on young people who possess the right skills and knowledge to contribute to the envisioned society. Good performance is the mantra of the entire plan as it is expected of both men and women in all aspects of life. Within the collectivistic traditions of the Middle East, good performance (as measured by grades) tends to be conceptualized not as the byproduct of competition among people but as a means that each person has to contribute to the collective to which that person belongs. In Saudi Arabia, the collective may be the tribe and, by extension, the aggregate of tribes that constitute the fabric of the country. In contrast, the neoliberal view of the economy may render performance a personal matter for which one’s relative standing in a group of peers carries some weight as jobs cannot be filled by all who want them. Consequently, grades may be prioritized as necessary tokens for personal advancement rather than being symbols of one’s contribution to the collective. In a society of contrasting values, such as Saudi Arabia, grades are likely to be seen through the lenses of both cultures without experiencing cognitive dissonance.

The initial goal of the present study is to organize students into three groups depending on their academic orientation [4]: those for whom attention to grades exceeds any consideration for learning (i.e., grade-oriented students), those for whom the opposite preference exists (i.e., learning-oriented students), and those for whom there is no preference. If the post-pandemic environment has conflated the two goals, most students will fall into the category of no preference (H1). The emotional state of such students may entail a relative amount of self-reported happiness and low writing anxiety as they see learning and grades as both valuable. Alternatively, if the pre-pandemic goal patterns have persisted in the post-pandemic environment, two hypotheses regarding the potential relationship between students’ predominant academic orientation and their emotional state can be formulated.

First, the degree of self-reported happiness of grade-oriented students will be lower than that of learning-oriented students (H2a). Furthermore, learning orientation will predict higher happiness, whereas grade orientation will predict lower happiness if not unhappiness (H2b). These hypotheses are based on pre-pandemic evidence that a learning goal orientation is typically correlated with indices of well-being, including positive affect, satisfaction, and engagement [1,57], whereas a grade orientation is less so. For instance, Black and Deci [58] found that students’ autonomous enrollment in a college-level organic chemistry course, which is an index of their internal motivation, was associated with students’ higher perceived competence, interest, and enjoyment. It was also associated with lower anxiety and less attention to grades during the course. Furthermore, it predicted whether or not the students withdrew from the course. Eison et al. [12] reported that learning-oriented students experienced less tension, frustration, and debilitating test anxiety and were highly self-motivated. In contrast, grade-oriented students were found to be anxious to meet conventional course standards, expressing concern for the grades they received. They exhibited poor study skills and high levels of debilitating anxiety. Vallade et al. [23] also reported that affective learning (i.e., students’ positive attitudes regarding a course, its content, and its instructor) was experienced to a lesser degree by grade-oriented students.

Second, in a challenging writing course, which demands mastering technical writing in a second language, grade-oriented students will experience more anxiety than learning-oriented students towards the task of writing (H3a). Furthermore, learning orientation will predict lower anxiety, whereas grade orientation will predict higher anxiety (H3b). These hypotheses are based on pre-pandemic evidence [1] that grade orientation is likely to be associated with the tendency to approach challenging situations with a threat framing, including negative emotions such as anxiety and fear. Instead, learning orientation is more likely to be associated with a challenge and opportunity framing. The latter is usually accompanied by constructive beliefs about the value of additional effort when setbacks occur.

Of course, in college, students choose to pursue different majors. A key distinction exists between STEM (science, technology, engineering, and mathematics) students and non-STEM students (business, law, etc.). Debate exists as to whether these two groups of students exhibit different characteristics pertaining to knowledge and attitudes [59,60]. Here, we ask whether they differ in academic orientation. Tani and Vines [21] found that law students (i.e., non-STEM majors) compared with students in other degree programs tend to be more focused on grades and academic attainment. Tani and Vines speculated that extrinsic motives may be linked to these students’ mental health issues. If these findings generalize to the broader field of non-STEM majors in the post-pandemic era, students in STEM fields will be found to be predominantly learning-oriented, whereas students in non-STEM majors will be found to be predominantly grade-oriented (H4). At this point, there is no evidence to predict that affective measures in STEM and non-STEM students will be differentially related to learning and grade orientations. Thus, it is reasonable to predict that if pre-pandemic data stand, a learning orientation will be linked to higher self-reported happiness and lower writing anxiety, irrespective of students’ selected majors. Instead, a grade orientation will be linked to lower self-reported happiness and higher writing anxiety (H2b and H3b).

## 4. Method

### 4.1. Participants

The participants were 287 female undergraduate students in the first year of their academic journey. They were enrolled in a course of the general education curriculum devoted to learning how to conduct and write about an empirical study. The course is the second of a sequence of writing-intensive courses. It is typically taken by freshmen during their second semester of university enrollment. Participants were all full-time students: 40.42% were STEM majors (e.g., computer science and engineering) and 59.58% were non-STEM majors (e.g., law and business). Their average age was 19.60 (range: 18 to 27 years). Students were Arabic-English bilingual speakers of Saudi Arabian nationality. The university in which they were pursuing their undergraduate education followed a US curriculum imparted in English and conformed to a student-centered pedagogy. Students’ English competency had been assessed before admission through standardized tests (e.g., TOEFL or IELTS). Students included in the sample were those who completed all measures described below (participation rate: 86.45%).

### 4.2. Materials and Procedure

All participants completed three questionnaires during the first weeks of the semester after having given informed consent. The Oxford Happiness Questionnaire (OHQ) of Hills and Argyle [25] was used to measure happiness largely from a hedonic perspective (subjective well-being) [61]. It consisted of 29 items. Pilot testing required a few minor changes in some of the items to ensure comprehension in the students of the selected population. A nine-point Likert scale, spanning from never (0) to always (8), was also adopted. The Cronbach alpha was 0.90.

The behavioral portion of the LOGO II questionnaire [4,62] consisted of eight learning-oriented statements and eight grade-oriented statements to be rated on a five-point Likert-type scale from never (0) to always (4). The Cronbach alpha was 0.77 for the learning-oriented scale and 0.62 for the grade-oriented scale.

Students also completed a revised version of the Second Language Writing Anxiety Inventory (SLWAIr) [63]. SLWAIr, which consisted of 22 items, was intended to assess students’ writing anxiety. Pilot testing required a few changes to the original scale driven by the need to adapt the scale to the student population of the selected university. Surface changes in the phrasing of some items ensured second-language speakers’ intuitive comprehension. For instance, the phrase “write English composition” was changed to “write assignments in English”. Participants were asked to report their answers on a five-point scale from strongly agree (+2) to strongly disagree (−2), with 0 serving as the neutral point. The Cronbach alpha was 0.93. The scale contained three sub-scales: somatic anxiety, behavioral avoidance, and cognitive anxiety. In the SLWAIr, somatic anxiety refers to one’s observation of the physiological effects of anxiety, such as the autonomic arousal that translates into an upset stomach, elevated heartbeat, sweating, and numbness. Behavior avoidance pertains to actions such as procrastination, evasion, and withdrawal. Cognitive anxiety denotes negative thoughts such as apprehension and negative expectations.

To ensure comfort, each item of every questionnaire was displayed in both English and Arabic. The Arabic text was developed by three scholars familiar with the constructs assessed by the questionnaires. Agreement among translators based on intelligibility guided the translation process. The guidelines of the International Test Commission for test translation and adaptation were followed [64].

Before students completed the questionnaires, they provided some demographic information, such as their age and major. ID numbers were used to link the data of the questionnaires and were immediately deleted afterward, ensuring confidentiality. The research complied with the guidelines of the Office for Human Research Protections of the U.S. Department of Health and Human Services in the treatment of participants in educational research. It was approved by the Deanship of Research at the institution where the data collection occurred.

## 5. Results

All inferential statistics were deemed significant at 0.05 level. Analyses were linked to the hypotheses they were intended to test.

### 5.1. Academic Orientation

For each student, the grade-oriented score was subtracted from the learning-oriented score to compute a preference score. A positive value indicated a learning preference, whereas a negative value indicated a preference for grades. A value of 0 illustrated neutrality. Among non-STEM students, 50.88% were learning-oriented and 49.12% were grade-oriented. Among STEM students, 63.79% were learning-oriented and 36.21% were grade-oriented. There were no participants with a 0 score (i.e., no-preference group). Thus, H1 was not supported. A Chi-Square Test indicated that there were considerably more learning-oriented students than grade-oriented students in STEM majors, whereas non-STEM students tended to be equally distributed between the two orientations [ꭓ^2^(1) = 4.68, *p* = 0.030]. This uneven pattern for majors partially supported H4. The next question to answer was whether there were also differences in happiness and writing anxiety.

### 5.2. Group Differences in Happiness and Written Anxiety

A Two-Step Cluster analysis with noise handling and Schwartz’s Bayesian Criterion yielded four groups of participants [average silhouette = 0.4, which signified a fair cluster quality]: STEM students with a preference for learning (STEM-LO), STEM students with a preference for grades (STEM-GO), non-STEM students with a preference for learning (non-STEM-LO), and non-STEM students with a preference for grades (non-STEM-GO). In Table 1, the mean and standard error of the mean of each subject group is displayed for self-reported happiness (as measured by OHQ) and second language writing anxiety (as measured by SLWAIr). The anxiety scores are organized into three dimensions: somatic anxiety, avoidance behavior, and cognitive anxiety.

An Independent-Samples Mann–Whitney *U*-Test was first used to compare the ranks of LO and GO students in each STEM and non-STEM group. STEM-LO students were happier [*U* = 1946.50, with the mean rank for LO equal to 63.80 and for GO equal to 49.15] and experienced less cognitive anxiety than STEM-GO students [*U* = 956.50, with the mean rank for LO equal to 50.43 and for GO equal to 72.73]. No differences in somatic anxiety and behavioral avoidance were observed between LO and GO STEM students [*U* < 1353.50, *ns*].

Non-STEM-LO students were also happier [*U* = 4898.00, with the mean rank for LO equal to 100.30 and for GO equal to 71.19]. They experienced less somatic anxiety [*U* = 2907.50, with the mean rank for LO equal to 77.42 and for GO equal to 94.89] and reported less behavioral avoidance [*U* = 2966.50, with the mean rank for LO equal to 78.10 and for GO equal to 94.18] than non-STEM-GO students. No difference in cognitive anxiety was observed between LO and GO non-STEM students [*U* = 3217.00, *ns*].

This pattern of results partially and indirectly supported H4 by illustrating that STEM and non-STEM students’ emotional states differed but only when a negative emotion, such as writing anxiety, was considered. H2a and H3a were also supported by the finding that in the post-pandemic era, LO students (irrespective of the chosen major) were happier and experienced overall less writing anxiety.

Testing of H2b and H3b entailed determining the extent to which in each major group (STEM and non-STEM) GO and LO scores would predict self-reported happiness and anxiety. In Table 2, Spearman correlation coefficients are reported with their respective coefficients of determination when correlations are deemed significant. Coefficients of determination illustrate the percentage of variance in each emotional state variable accounted for by either GO or LO variables.

For STEM students, LO predicted their happiness and GO predicted their unhappiness. GO scores predicted higher somatic and cognitive anxiety, whereas LO scores predicted less avoidance behavior and cognitive anxiety. For non-STEM students, LO scores predicted their happiness, lower somatic anxiety, and less avoidance behavior. GO scores did not predict any emotional state. This pattern of relationships only partially supported H2b and H3b. Coefficients of determination, however, were rather small, suggesting that the link between academic orientation and emotional state was rather weak.

## 6. Discussion

The findings of the present study can be summarized in four points. First, in the post-pandemic environment, the goals of learning and getting good grades are not conflated, which would lead most students to fall into the category of no preference (as predicted by H1). On the contrary, students had a preference, although the two orientations coexisted to a certain degree. When participants were asked to explain during debriefing their views of grades, learning-oriented and grade-oriented students produced starkly different answers. Self-identified learning-oriented students saw grades as a weak form of feedback. They found comments made by instructors on assignments or tests more useful and grades as important but of little practical utility if corrective measures were to be considered. Self-identified grade-oriented students saw grades as measures of their self-concepts and grading as a potential threat. Comments made by instructors on their assignments or tests were received defensively, often leading to questions about how specific comments were linked to the grades received. Students’ focus was on the points they lost. Frequent grade inquiries were driven by the expectation of a full mark.

Second, for both STEM and non-STEM majors, the degree of self-reported happiness of grade-oriented students was lower than that of learning-oriented students (as predicted by H2a). Learning orientation predicted higher happiness, whereas grade orientation predicted unhappiness but only in STEM students. Therefore, H2b was only partially supported given the null results of grade orientation for non-STEM students. The clear-cut pattern of relationships exhibited by STEM students might be linked to the challenges that materials and instruction of STEM curricula pose to students. Scientific materials, such as textbooks and articles, are often written in a technical language with concept-dense and abstract contents [65]. Learning is similarly challenging as it tends to focus on technological innovation for the design and application of solutions to complex contextualized problems [66]. Furthermore, in STEM disciplines, high-stakes tests [67] are often used to assess performance that rely on students’ understanding of the complexities of the curriculum. Thus, the STEM ecosystem could be conceived as putting more directly a premium on students’ learning orientation as a conduit to desirable performance. Benefits to students’ emotional states arise as the adoption of the latter is likely to produce desirable levels of academic attainment.

Third, in a challenging writing course, which demanded mastering technical writing in a second language, grade-oriented students experienced more anxiety than learning-oriented students towards the task of writing (H3a). However, there were differences in the type of anxiety experienced by grade-oriented students in STEM and non-STEM majors. Grade-oriented STEM students suffered more from cognitive anxiety (apprehension and negative expectations), whereas grade-oriented non-STEM students suffered more from somatic anxiety (i.e., autonomic arousal that translates into an upset stomach, elevated heartbeat, sweating, and numbness) and behavioral avoidance (procrastination, evasion, and withdrawal). Furthermore, for STEM students, learning orientation predicted lower cognitive anxiety and behavioral avoidance, whereas grade orientation predicted higher somatic and cognitive anxiety. Instead, for non-STEM students, learning orientation predicted lower somatic anxiety and behavioral avoidance, whereas grade orientation did not predict any of the forms in which anxiety can manifest itself. Therefore, H3a and H3b were only partially supported, further reinforcing the notion that STEM and non-STEM students are different.

Fourth, students in STEM fields were predominantly learning-oriented, whereas students in non-STEM majors were evenly divided between grade-orientation and learning-orientation. This pattern partially supported H4. It also provided further support for the notion that the STEM ecosystem, compared to the non-STEM ecosystem, reinforces more directly students’ learning orientation.

Overall, our findings are consistent with those that have reported a relationship between subjective well-being and learning orientation. Subjective well-being includes a broad category of phenomena from global emotional responses and reports of life satisfaction to more localized responses, such as academic satisfaction. Our study focuses on happiness (a positive emotion that defines well-being) and writing anxiety (a task-specific negative emotion). Sánchez-Cardona et al. [68], instead, targeted academic satisfaction, defined as a student’s positive emotional state resulting from the appraisal of educational experiences. They reported a direct link between learning orientation and academic satisfaction. Roebken [69] also found that a learning orientation is related to students’ greater satisfaction with their academic experiences and higher overall performance. Debicki et al. [16] studied core self-evaluations, defined as students’ assessments of their worthiness, competence, and ability. They found a positive association between learning orientation and core self-evaluations. Thus, notwithstanding the particular type of positive emotional response examined in each of these studies, and in ours, learning orientation remained the consistent counterpart.

### 6.1. The Socio-Cultural Context of Academic Orientation

To understand the relationship between academic motivation and affective states in Middle Eastern students, the coexistence of collectivistic and individualistic motives is to be acknowledged. That is, in a society of contrasting values, such as Saudi Arabia, grades are seen through collectivistic and individualistic lenses without experiencing cognitive dissonance. Indeed, in debriefings, self-identified grade-oriented students described grades as a tangible currency through which young people can contribute to the collective to which they belong. Yet, grades were not only seen through the collectivistic lenses of participants’ traditional culture. Grades were also viewed through the individualistic lenses of the Western culture, whereby grades were tokens for greater personal rewards (a diploma, a job, etc.), raising the fear of competition for scarce resources. In either case, it is not surprising that the negative state of mind that has been often associated with grade orientation was also found in Middle Eastern students. It arose from framing situations that entail the measurement of performance as threats.

Happiness as an index of well-being may also be linked to different goals (i.e., what makes human beings tick) depending on the cultural setting [70]. An individualistic credo sees well-being in terms of achievement, autonomy, pleasure, and stimulation, thereby defining happiness as the maximization of positive life events, even at the expense of others. Instead, a collectivistic credo sees well-being as social harmony and the fulfillment of role obligation. Even negative life events may be viewed positively as they promote social support. In debriefings, these two conceptualizations of happiness emerged when students were asked to explain their views of happiness. When prodded regarding the likely discrepancy between these two conceptualizations, students claimed that both views can be pursued by a person without experiencing contradictions. Thus, the culturally mixed socio-cultural context in which Middle Eastern students are embedded allows these two contrasting conceptualizations to exist side by side (as per evidence collected from debriefing). Students may use one or the other depending on the circumstances.

### 6.2. Limitations and Future Directions

The present study has limitations that need to be addressed in future research. First, the sample of participants included female students at the start of their academic careers. We were unable to access a comparable sample of male students due to the campus being divided by gender. Although counselors at the selected university often mentioned female students as being the most vulnerable to anxiety, it is unclear whether the same pattern of results would be found in male students. Gender differences may exist due to socio-economic forces. Namely, female college students are the fulcrum of Saudi Arabia’s 2030 Vision. As such, they are faced with considerable pressure to succeed academically. For male college students, academic success is not a novel obligation. Thus, their orientation and affective state might exhibit a similar but attenuated pattern. Second, the information gathered here entailed self-reports collected in the classroom, which may exhibit biases [71]. Third, the extent to which current findings can be generalized to other student populations requires scrutiny. For instance, the Middle Eastern participants of our post-pandemic study displayed the coexistence of grade and learning orientation with a preference for either. It is unclear whether students in the Global North currently exhibit the same pattern of coexistence of orientations and the same relationships with affective states. Fourth, there may be additional ingroup differences that we were unable to test within STEM students or non-STEM students depending on their particular major. Fifth, although academic orientation was found to be related to emotional states, it accounted for a very small portion of the variance in such states. Weak relationships suggest that other factors, untested here, might yield a more substantial contribution to students’ emotional states in STEM and non-STEM fields. Sixth, the extent to which performance may be shaped by academic orientation has remained untested. Yet, pre-pandemic evidence does not suggest that performance differences as a function of academic orientation would be found [7]. The reason might be that in academia, regardless of the orientation used to approach classes, grades matter. Six, our study is correlational in nature. It is not an experiment in which we manipulated variables under controlled conditions to test cause–effect relationships. In our study, variables were some selected properties of the participants. Thus, academic orientation may be assumed to affect emotional states broadly (e.g., happiness) or situationally (e.g., writing anxiety). It is also possible that students’ emotional states make one academic orientation dominant over the other. Alternatively, it may be other variables, such as particular characteristics of the academic setting, which are responsible for the link between academic orientation and emotional state.

### 6.3. Pedagogical and Theoretical Implications

Within the literature on achievement goals, there have been different labels used for largely the same constructs. Of interest here is that achievement goal orientations can be defined by their focal purpose: mastery or performance [72,73,74]. Labels such as mastery or process goals and learning orientation refer to a learner’s aspiration to improve competencies, whereas performance goals and grade orientation refer to the learner’s aim of demonstrating competence often in tangible and measurable forms (e.g., grades). Our findings exist within this literature. They suggest that the assessment of dispositions is a valuable tool for not only understanding college students but also developing effective instruction. Specifically, interventions for at-risk students may be more or less impactful depending on the extent to which individual differences are acknowledged. Indeed, for students with a preference for grade orientation, academic difficulties will need to address both negative emotional states and their approach to classes, mostly to weaken the threat approach with which such students see difficulties. Dismantling the threat reaction and substituting it with a challenge reaction is desirable. Instead, for students with a preference for learning, academic difficulties may be more targeted to the particular skill and knowledge that is deficient, perhaps helping students find alternative study methods or offering practice opportunities. In either group, the goal is not only to foster academic success but also to promote well-being.

It is important to note here that interventions may target structural factors, curricula, and instruction, or have as a primary focus the promotion of students’ well-being and psychological resources [75,76]. Yet, the two approaches serve the common goal of ensuring optimal learning under optimal conditions. Thus, understanding the dispositions that are linked to students’ engagement, satisfaction, and academic success is key to designing academic curricula and instructional modes that promote learning as well as well-being in all students [75,77,78].

The broader theoretical implication of our findings is that students’ ways of responding to challenging situations (e.g., college classes) are related to their emotional states. As such, our findings are consistent with the theory of goal orientation [1]. Within this theory, the notion of academic orientation corresponds to the broader construct of academic mindsets. The concept of learning orientation fits the growth mindset [79], according to which intellectual abilities can be developed over time through sustained effort, especially when challenges and setbacks are encountered. A learning orientation is known to promote the use of adaptive behaviors, which are key to self-regulated learning. The latter refers to students’ regulation of thoughts, feelings, and actions to attain learning goals. Not surprisingly, the coexistence of learning orientation and self-regulated learning in a variety of learners is often portrayed as beneficial to academic performance [78]. Furthermore, interventions targeting emotional regulation [76] are seen as making students more able to cope with challenging situations (e.g., exams), benefiting their emotional states as well as performance. Emotional regulation may be particularly important for performance after a disruptive event, such as the pandemic, when readaptation to on-campus life adds to the ordinary challenges of college demands [80]. Consideration of individual differences in grade orientation appears to be an effective way of identifying students who may benefit from interventions targeting emotional regulation.

## 7. Conclusions

To succeed in college and beyond, students in today’s educational landscape must be able to adequately respond to a variety of academic demands. Demands often stem from curricula and instruction that do not take into consideration individual differences in dispositions and emotional states. In the Middle Eastern culture of Saudi Arabia, individualistic forces of Western import and the collectivistic ethos of local communities join forces in promoting academic and professional success for the country that Saudi Arabia wishes to become [81,82]. In this context, youth’s success in STEM fields is a priority. The stakes of failure are higher for women who have only recently gained access to gender-equitable education. Agency and independence are linked to educational attainment. Yet, for both men and women, the key question is how they conceptualize success in their field of choice. Is success equated to competence and perhaps an approach to learning that continues into a profession? Is success a quantifiable marker, such as GPA, which opens the door to scholarships and jobs? The two are inextricably linked together, albeit our study’s findings show that each student prioritizes one over the other. Furthermore, in our study, learning orientation was linked to more favorable emotional states. Most importantly, students who chose STEM were more likely to prioritize learning over grades than non-STEM students. This pattern is good news for a country that has invested substantial resources into restructuring its fossil fuel economy into a knowledge-based one [83].

## Figures and Tables

**Table 1:** Descriptive statistics.

	LO	GO	Happiness	Somatic Anxiety	Avoidance Behavior	Cognitive Anxiety
STEM-GO *n* = 42	1.18 (0.10)	2.19 (0.10)	4.80 (0.11)	−0.44 (0.16)	−0.80 (0.11)	+0.17 (0.10)
STEM-LO *n* = 74	2.30 (0.07)	1.33 (0.06)	5.32 (0.09) *	−0.78 (0.12)	−1.04 (0.09)	−0.32 (0.08) *
Non-STEM-GO *n* = 84	1.31 (0.07)	2.00 (0.06)	4.92 (0.08)	−0.30 (0.14)	−0.60 (0.09)	−0.03 (0.08)
Non-STEM-LO *n* = 87	2.43 (0.06)	1.42 (0.06)	5.59 (0.11) *	−0.61 (0.19) *	−0.90 (0.12) *	−0.19 (0.12)
Range	0–4		0–8	−2–+2	−2–+2	−2–+2

Note: Significant comparisons between GO and LO for each college major group are labeled with an asterisk. * significant differences.

**Table 2:** Spearman correlation coefficients.

STEM	Happiness	Somatic Anxiety	Avoidance Behav.	Cognitive Anxiety
GO	−0.19 * 3.61%	+0.22 * 4.84%	+0.07	+0.22 * 4.84%
LO	+0.22 * 4.84%	−0.07	−0.24 * 5.76%	−0.27 *
Non-STEM				
GO	−0.13	+0.13	+0.07	+0.09
LO	+0.28 * 7.84%	−0.19 * 3.61%	−0.21 * 4.41%	−0.05

Note: Significant correlations are labeled with *.

## Data Availability

Data are available upon request.

## Appendix A

A selected group of instructors (*n* = 15) who taught general education courses were asked to describe their students, how they taught, and the challenges they encountered. Responses to these open-ended questions constituted narrative records written during or immediately after each interaction. Records were organized by two independent raters into themes [84] if a majority of the instructors mentioned them. In response to these open-ended questions, all instructors described themselves as adopting a student-centered pedagogical approach. They also mentioned that their attempts to focus students’ attention on learning were either welcomed or resisted depending on the degree to which students emphasized grades [85]. Students who prioritized grades were reported as consistently questioning the distribution of points acquired in assignments and tests. Such students paid little attention to remedial feedback if it was not accompanied by opportunities to redo and regrade their work. They were also described as expressing behavioral manifestations of anxiety before, during, and after tests, as well as before and after assignments were graded. Instructors noted that students who prioritized grades tended to forecast a doom scenario for their performance. An array of negative emotions plagued these students, all focused on the threat that a less-than-perfect mark would inflict on their present and future lives. These patterns, which had been documented before the pandemic [7], did not appear to change after the pandemic.
